# Hemothorax After Retroclavicular Approach to the Infraclavicular Region in a Critically Ill Patient: A Case Report

**DOI:** 10.7759/cureus.49876

**Published:** 2023-12-03

**Authors:** Sara Ribeiro, André Pombo, Neusa Lages, Carlos Correia, Carla Teixeira

**Affiliations:** 1 Anesthesiology, Intensive Care and Emergency Department, Centro Hospitalar Universitário de Santo António, Porto, PRT; 2 Anesthesiology Department, Hospital Narciso Ferreira, Santa Casa da Misericórdia de Riba de Ave, Braga, PRT

**Keywords:** upper extremity, trauma, regional anesthesia, pain management, hemothorax, delirium, critical care, case report, brachial plexus block

## Abstract

The retroclavicular approach to the infraclavicular region (RAPTIR) is a recently described locoregional technique for upper limb analgesia that offers advantages over the classic infraclavicular block. RAPTIR is considered an effective and easy-to-perform block associated with few complications and better patient comfort.

We present a case of a critically ill patient with thoracic and upper limb trauma. Despite multimodal analgesia, the patient developed delirium and experienced suboptimal pain control. An ultrasound-guided continuous RAPTIR block was performed, resulting in improved pain scores and delirium control. Twenty-four hours post block, the patient presented with dyspnea and chest pain, leading to the diagnosis of hemothorax. Chest computed tomography angiography revealed no vascular damage. The perineural catheter was removed 48 hours after its placement and the patient had a satisfactory recovery without long-term complications.

The RAPTIR requires the needle to pass underneath the clavicle’s acoustic shadow, putting the structures beneath the clavicle at risk of injury. Cadaver studies have raised concerns about potential vascular complications of the RAPTIR in a noncompressible location. This case highlights, for the first time, a rare but serious complication of the RAPTIR, demonstrating the potential risks of passing the needle through a blind spot.

## Introduction

Critically ill patients commonly experience moderate to severe pain at rest and during standard care procedures [1]. Pain leads to various detrimental physiological changes and is a risk factor for the onset of delirium [2,3]. The prevalence of delirium in intensive care units (ICUs) can reach up to 25%, and it is linked to negative short-term and long-term outcomes [3-6].

Current clinical practice guidelines recommend regular assessments of pain and delirium using validated scales. Nonetheless, pain continues to be inadequately assessed, particularly in high-risk patients, such as critically ill individuals [1]. On the other hand, pain treatment in the ICU setting faces unique challenges, as acute organ dysfunction can affect the pharmacokinetics and pharmacodynamics of systemic analgesia, while analgesics themselves can worsen or contribute to organ dysfunctions. Opioids remain the cornerstone of pain management in ICUs but are associated with delirium and respiratory depression, among other undesirable effects. Regional anesthesia (RA) techniques can complement systemic analgesic approaches by providing excellent pain relief and reducing the need for opioids.

We report a case that highlights the importance of RA in the effective management of pain and delirium in a critically ill patient with superior limb trauma. A continuous infraclavicular brachial plexus nerve block (ICB) was performed using a retroclavicular approach to the infraclavicular region (RAPTIR). The RAPTIR, initially described by Hebbard and Royse, offers excellent in-plane needle visualization as the angle of the needle insertion is parallel to the probe [7]. Charbonneau later reported a 90% success rate for sensory block and a 96% success rate for surgical outcomes [8]. Today, RAPTIR is regarded as an effective and easy-to-perform block with few complications.

## Case presentation

Written informed consent was obtained from the patient and his family for the publication of this case report. We report a case of a 64-year-old man who experienced a farm tractor rollover accident, resulting in superior limb and thoracic trauma. The major injury involved an avulsion of the right forearm with a complete vasculo-nervous section at the cubital fossa and a comminuted fracture of the elbow. Additionally, three fractured ribs on the right were identified. Past medical records revealed a history of long-term hypertension. It is worth noting that the patient tested positive for SARS-CoV-2 upon admission to the hospital.

**Figure 1:**
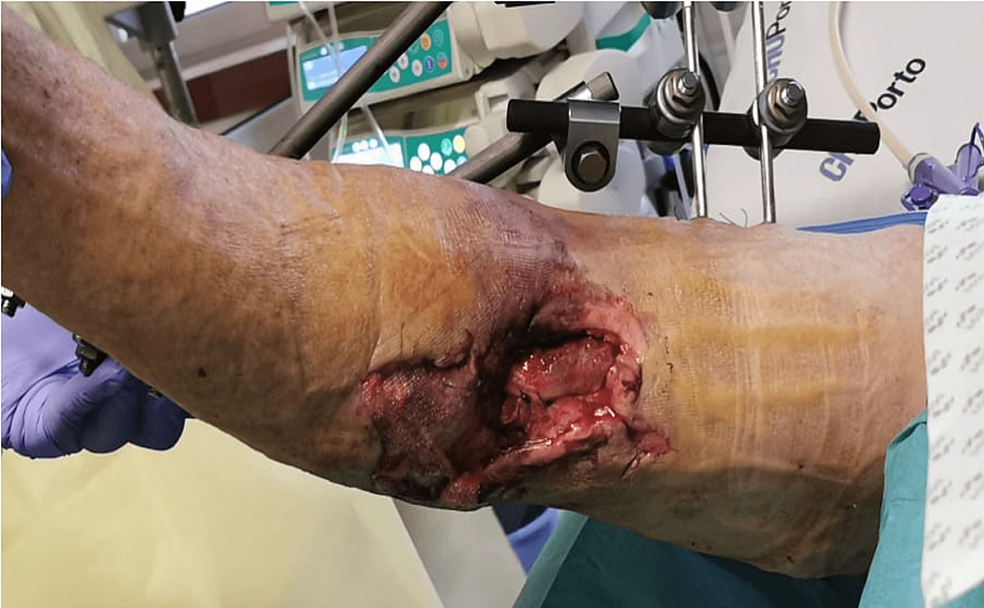
Upper limb trauma Upper limb trauma showing tissue damage during surgical dressings.

Brachial artery reconstruction with a great saphenous vein graft was immediately performed. After the surgery, the patient was admitted to the ICU, where several major problems were addressed. Firstly, post-traumatic hemorrhagic shock was managed, requiring blood product transfusion and a noradrenaline infusion. Later, the patient developed respiratory insufficiency due to thoracic trauma and the contribution of coronavirus disease. On the 10th day, the patient was diagnosed with mixed delirium according to the Confusion Assessment Method in the ICU (CAM-ICU). The treatment of delirium involved a combination of non-pharmacological methods and pharmacological intervention, including dexmedetomidine (maximum = 1.4 mcg/kg/h), quetiapine (400 mg daily), oxazepam (50 mg every eight hours), and thiamine (100 mg daily), but yielded suboptimal results. Furthermore, postoperative pain control proved to be a significant challenge. After vascular reconstruction, the right arm wound required daily surgical dressings, which were associated with the highest pain scores (Figure 1). Pain was regularly assessed using Behavioral Pain Scale (BPS) or Numerical Rating Scale (NRS) and treated with systemic analgesia, including paracetamol (1 g every eight hours), metamizole (2 g every eight hours), ketorolac (30 mg every 12 hours), tramadol (100 mg every eight hours), fentanyl (50 mcg/h), and pregabalin (100 mg every 12 hours). However, the patient's pain scores at rest were NRS of 5-7/BPS of 4-6 and NRS of 7-9/BPS of 8-10 during surgical dressings. Moreover, when the right arm was mobilized for positioning and during the changing of surgical dressings, the patient presented with tachycardia and hypertension.

**Figure 2:**
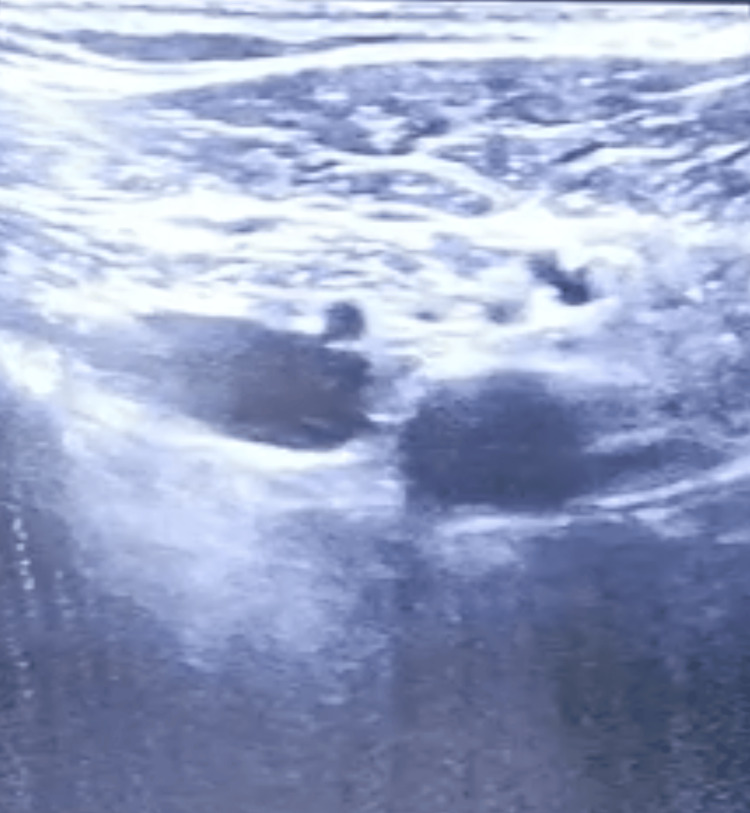
Ultrasound image of the RAPTIR The needle shaft is located posteriorly to the axillary artery after emerging from the acoustic shadow of the clavicle. RAPTIR: retroclavicular approach to the infraclavicular region.

**Figure 3:**
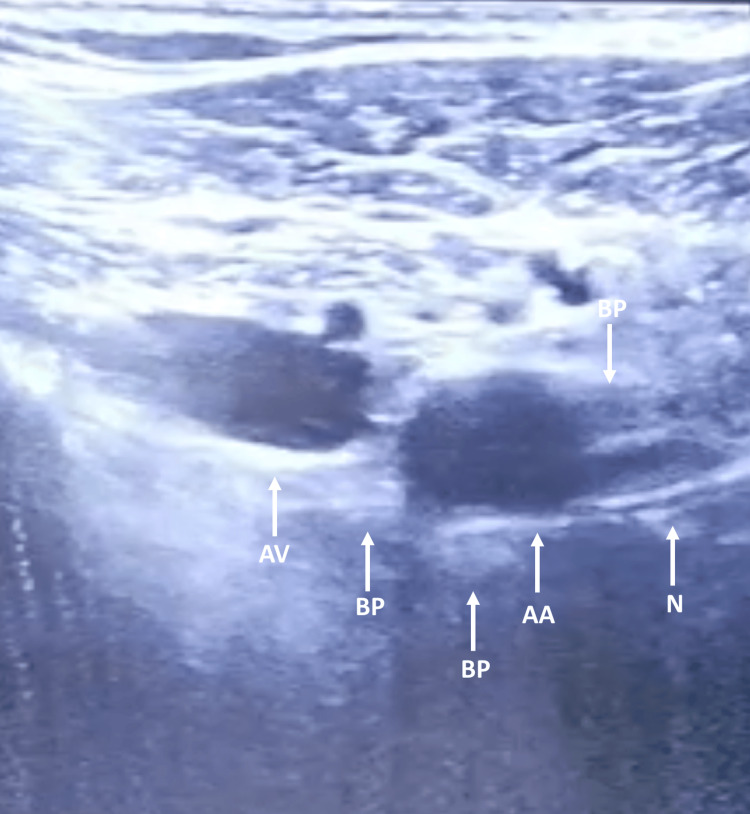
Visualization of ultrasound landmarks for performing the RAPTIR RAPTIR: retroclavicular approach to the infraclavicular region; AV: axillary vein; AA: axillary artery; BP: cords of the brachial plexus; N: needle shaft.

A continuous ICB using the RAPTIR technique was performed under ultrasound (US) assistance and peripheral nerve stimulation (PNS) guidance. Following the successful visualization of neurovascular structures, an echogenic needle was employed to administer 20 ml of 0.5% ropivacaine posteriorly to the axillary artery, and a multiorifice catheter was easily advanced 4.5 cm beyond the needle tip (Figures 2, 3). A 0.2% ropivacaine infusion was initiated at a rate of 5 ml/h using an elastomeric pump, and a 20 ml bolus of the same ropivacaine solution was prescribed prior to the daily change of surgical dressings. The patient experienced significant pain relief, rating 3-4 points on BPS at rest and 5-8 points during surgical dressings. Furthermore, we verified a continuous improvement in the patient's hypertension and tachycardia profile. According to CAM-ICU, delirium was also better controlled.

**Figure 4:**
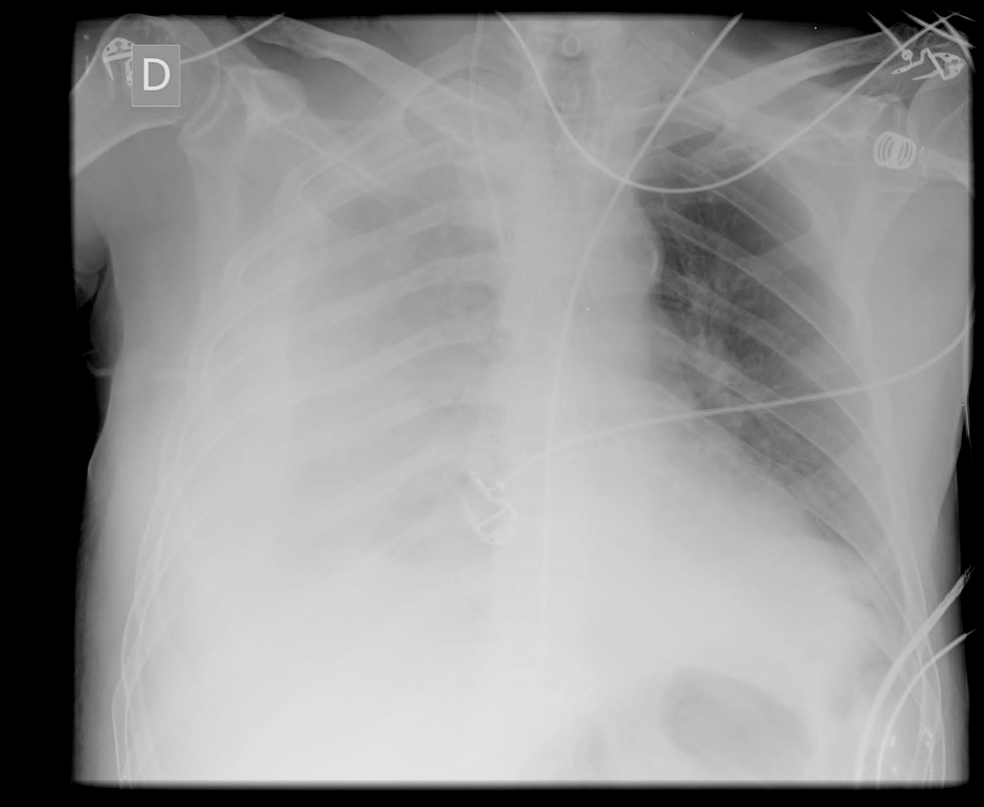
Thoracic radiography 24 hours post block

Twenty-four hours after the block, the patient developed dyspnea and right-sided chest pain accompanied by tachycardia and hypotension. A chest radiograph showed a whiteout appearance in the right lung field without tracheal deviation leading to the diagnosis of hemothorax (Figure 4). A right-sided chest tube was immediately inserted and approximately 1350 ml of dark red-colored blood was drained. The hemothorax was considered a complication of ICB, as no other plausible cause could be identified at that moment. Chest computed tomography angiography did not reveal any leakage of contrast media. The perineural catheter was removed 48 hours after its placement. The patient experienced a satisfactory recovery and no long-term sequelae were observed.

## Discussion

We decided to perform an RA technique aiming at pain control and delirium reduction. Our goal was to execute a continuous brachial plexus block to target the nociceptive stimuli originating from the cubital fossa. We considered an infraclavicular approach as it would offer the advantages of easy catheter fixation with minimal phrenic nerve block, which was specifically important due to the patient’s thoracic trauma and coronavirus disease. However, the classic coracoid infraclavicular approach has a steep insertion angle of the needle, which can pose difficulties, especially in uncooperative patients. Therefore, we opted to perform an ICB using the alternative RAPTIR [7-10].

Performing RA in the specific setting of ICUs presents unique challenges as techniques may be more difficult to perform due to the lack of patient cooperation and the presence of tissue damage or edema. In this case, the block was performed after sedation and under the guidance of US and PNS. We experienced no trouble identifying the neurovascular structures or the local anesthetic dispersion. However, it is important to mention that the RAPTIR requires the needle to pass through the acoustic shadow of the clavicle, putting the structures beneath the clavicle at risk of injury. A recent cadaver study investigating the structures encountered by the needle during the RAPTIR showed that the suprascapular nerve is consistently found in the needle’s path beneath the clavicle and reported a case where the needle shaft was in close contact with the supraclavicular vein, raising concerns about potential vascular complications of the RAPTIR [11].

To our knowledge, there are no previous reports of hemothorax after the RAPTIR. In this case, the rate of blood accumulation in the pleural cavity seemed to be low as it took 24 hours for the hemothorax to become clinically evident, suggesting a venous source as the cause of bleeding. Moreover, the dark red color of the drained blood and the absence of vascular damage on chest computed tomography angiography were consistent with the spontaneous control of venous bleeding. Although the exact injured vessels were not identified, we contemplated the hypothesis of supraclavicular vessels or subclavian vein injury during the needle's path beneath the clavicle. It is worth noting that while in the previously mentioned cadaver study, the subclavian vein was not considered to be one of the structures at risk of injury, limitations of cadaveric models, such as lung deflation, may affect the accuracy of the representation of potential RAPTIR complications in living patients [11].

Another key consideration is that any complication of RA, such as infection, bleeding, or hemopneumothorax, can have more severe consequences for critically ill patients, as their physiologic reserve is already compromised [7]. In the described case, the patient suffered hemorrhagic and obstructive shock, requiring the insertion of a thoracic chest tube, blood transfusions, and the administration of vasoactive drugs. While there were no long-term sequelae in this specific case, this complication made us rethink our approach and realize that performing an alternative technique such as the classic infraclavicular or costoclavicular block would have been a better choice. In fact, although randomized controlled trials comparing the RAPTIR with the classic coracoid approach did not find statistically significant differences in terms of complications, the number of patients included in these studies may not be sufficient to establish the RAPTIR as an equally safe block [12,13].

## Conclusions

In conclusion, we have presented, for the first time, a potentially life-threatening complication of the RAPTIR. With this case, we aim to draw attention to the dangers of passing the needle through a blind spot and raise the question of whether the ease of performance and good results achieved with the RAPTIR outweigh its potential risks.
